# Detection of Bioavailable Cadmium by Double-Color Fluorescence Based on a Dual-Sensing Bioreporter System

**DOI:** 10.3389/fmicb.2021.696195

**Published:** 2021-09-16

**Authors:** Chang-ye Hui, Yan Guo, Jian Wu, Lisa Liu, Xue-qin Yang, Xiang Guo, Ying Xie, Juan Yi

**Affiliations:** ^1^Department of Pathology & Toxicology, Shenzhen Prevention and Treatment Center for Occupational Diseases, Shenzhen, China; ^2^National Key Clinical Specialty of Occupational Diseases, Shenzhen Prevention and Treatment Center for Occupational Diseases, Shenzhen, China; ^3^Lewis Katz School of Medicine, Temple University, Ambler, PA, United States

**Keywords:** whole-cell biosensor, fluorescent signal, cadmium detection, CadR, CadC

## Abstract

Cadmium (Cd) is carcinogenic to humans and can accumulate in the liver, kidneys, and bones. There is widespread presence of cadmium in the environment as a consequence of anthropogenic activities. It is important to detect cadmium in the environment to prevent further exposure to humans. Previous whole-cell biosensor designs were focused on single-sensing constructs but have had difficulty in distinguishing cadmium from other metal ions such as lead (Pb) and mercury (Hg). We developed a dual-sensing bacterial bioreporter system to detect bioavailable cadmium by employing CadC and CadR as separate metal sensory elements and eGFP and mCherry as fluorescent reporters in one genetic construct. The capability of this dual-sensing biosensor was proved to simultaneously detect bioavailable cadmium and its toxic effects using two sets of sensing systems while still maintaining similar specificity and sensitivity of respective signal-sensing biosensors. The productions of double-color fluorescence were directly proportional to the exposure concentration of cadmium, thereby serving as an effective quantitative biosensor to detect bioavailable cadmium. This novel dual-sensing biosensor was then validated to respond to Cd(II) spiked in environmental water samples. This is the first report of the development of a novel dual-sensing, whole-cell biosensor for simultaneous detection of bioavailable cadmium. The application of two biosensing modules provides versatile biosensing signals and improved performance that can make a significant impact on monitoring high concentration of bioavailable Cd(II) in environmental water to reduce human exposure to the harmful effects of cadmium.

## Introduction

Cadmium occurs naturally and is usually found in volcanic soils. Cadmium also occurs as a pollutant originating from human activities, which is released predominantly from mine drainage, industrial processes, and inappropriate disposal of cadmium-containing batteries (Meena et al., 2018). Cadmium and its compounds are well-known as a food contaminant. Due to its enrichment, cadmium is carcinogenic to humans (World Health Organization, 2010; Mie et al., 2017; Genthe et al., 2018). Non-cellular biosensors based on interaction between DNA and proteins with cadmium have been developed (Ebrahimi et al., 2018; Lee et al., 2019; Xue et al., 2020). Although these existing physical and chemical techniques for cadmium detection are sensitive and accurate, they cannot provide information on both its bioavailability and its toxic effects on exposed organisms. Whole-cell biosensors are a better option and can assess the bioavailability and the toxic effects of heavy metal in the environment (Wanekaya et al., 2008; Gupta et al., 2019).

Many heavy metals such as cadmium do not have a biological role and cause extreme toxicity to living organisms, including microorganisms. Therefore, prokaryotes have evolved a toxic metal homeostasis system regulated at the transcriptional level (Jung and Lee, 2019). SmtB/ArsR family and MerR family are two major metalloregulatory protein families, which were well characterized for the transcriptional regulation of detoxifying genes in response to heavy metal ions (Ma et al., 2009; Waldron et al., 2009). Using these two kinds of metalloregulatory proteins as the sensory elements, a large number of heavy metal biosensors have been developed over the past decades. Based on engineered microorganisms, biosensing constructs were assembled to produce both qualitative and quantitative signals after exposure to heavy metals (Bereza-Malcolm et al., 2015; Kim et al., 2018). The *zntA* on the chromosome of *Escherichia coli* (Brocklehurst et al., 1999), *cadC* on the pI258 plasmid of *Staphylococcus aureus* (Endo and Silver, 1995), and *cadR* in *Pseudomonas putida* 06909 (Lee et al., 2001) were generally used as the Cd(II)-sensing transcription regulators to assemble biosensor constructs.

The *zntA* promoter was fused to a promoterless *luxCDABE* operon to assemble a metal-inducible construct. Specifically, the metals Cd(II), Pb(II), Hg(II), and Zn(II) induced the luciferase activity of the construct in recombinant *E. coli* with chromosomally encoded ZntR (Riether et al., 2001). When the zinc regulatory gene *zntR* and the *zntA* promoter was fused with the promoterless *gfp*, the resultant biosensor was significantly induced by 0.045–35.7 μM Cd(II), 46–12,307 μM Zn(II), and 0.005–0.6 μM Hg(II) (Gireesh-Babu and Chaudhari, 2012). The whole-cell biosensor using the promoter region of zinc-inducible *znt* operon showed specific and sensitive responses to Cd(II) rather than Zn(II) under specific experimental conditions (Yoon et al., 2016a). However, the ZntR-based biosensor was further demonstrated to respond to Zn(II) with a similar level to Cd(II) when induced with a much higher concentration of Zn(II) (Wang et al., 2013).

The *gfp* reporter was cloned into the *cadCA* operon from *Staphylococcus aureus* plasmid pI258. The developed biosensor showed responses to Cd(II) within the range 0.09–0.45 μM, with responses slightly interfered by Pb(II) and Zn(II) (Kumar et al., 2017). The CadC-T7 circuit was used to develop a highly sensitive biosensor, which could be induced by Cd(II) and Pb(II) (Kim et al., 2016).

Cloning a single-signal output element CadR-GFP into a broad-host-range plasmid led to the assembly of a multiplex cadmium biosensing construct. Several Gram-negative bacteria including *Pseudomonas*, *Shewanella*, and *Enterobacter* were transformed with the biosensing construct, and then, the resultant whole-cell biosensors became responsive to Cd(II) levels ranging from 0.09 to 90 μM, as well as to several other heavy metals, including As(III), Hg(II), and Pb(II) at similar concentrations (Bereza-Malcolm et al., 2017). The *cadR* and the divergent *cadR* promoter was used as the sensory element to develop the Cd(II) biosensors with red fluorescence, green fluorescence, and β-galactosidase as signal outputs. Engineered *E. coli* responds strongly to Cd(II) and weakly to Hg(II). Reporter signals of engineered *E. coli* cultured in M9 medium were shown to increase within Cd(II) level ranges of 0.1–3.125 μM (Guo et al., 2021).

Although a substantial number of cadmium whole-cell biosensors have been developed, their performances, and in particular their specificities, are not satisfactory as they respond to Cd (II), Hg(II), and Zn (II). In order to improve the specificity of whole-cell biosensor for cadmium, the amino acid located in the metal-binding loop of ZntR was changed, and thus, the resultant biosensors showed responses specific to only Cd(II) and Hg(II) (Kang et al., 2018). By truncating 10–21 amino acids from the C-terminus of CadR, the responses of the resultant biosensor toward Zn(II) and Hg(II) were significantly decreased (Tao et al., 2013). Several Cd-specific MerR mutants were generated by directed evolution. The function of these mutations were assessed using a construct in which MerR mutant controlled the production of the reporter GFP and luciferase *via* the divergent *mer* promoter. These biosensor constructs were successful and did not generate responses to Hg(II) and Zn(II) (Hakkila et al., 2011).

Integration of two sets of sensing elements into a single bacterial strain has been previously demonstrated as a useful method to develop multiple-signal-output biosensors, to develop multiple functional engineered strains (Maruthamuthu et al., 2015, 2017) and to enable simultaneous detection of several heavy metals (Yoon et al., 2016b). In this study, a dual-sensing bacterial bioreporter system employing the CadR and CadC as sensory elements and the mCherry and eGFP as reporters was developed. The performance of the designed biosensors including selectivity and sensitivity was investigated. The biosensing characteristics and complementary advantages of two sensory elements were successfully integrated into one engineered bacterial strain.

## Materials and Methods

### Bacterial Strains, Plasmids, and Agents

The bacterial strain and plasmids used in this study are listed in Table 1. *E. coli* TOP10 was used as a host strain for both cloning and biosensing. Recombinant strains were routinely cultured at 37°C in Luria-Bertani (LB) broth (1% tryptone, 0.5% yeast extract, and 1% NaCl) or on LB agar plates supplemented with 50 μg/ml ampicillin. All reagents and kits for molecular cloning were purchased from Sangon Biotech (Shanghai, China). Oligonucleotides and synthetic DNA were obtained from Sangon Biotech. All chemicals used in this work were of analytical grade and were purchased from Sigma-Aldrich (St Louis, MO, United States). Stock solutions of CdCl_2_, HgCl_2_, Pb(NO_3_)_2_, ZnSO_4_, CuSO_4_, MnSO_4_, NiSO_4_, CaCl_2_, and MgCl_2_ were freshly prepared with doubly distilled water. DNA sequencing to verify correctly assembled constructs was performed by Sangon Biotech.

**TABLE 1:** Bacterial strains and plasmids used in this study.

Strain and vectors	Genotypes or description	References
**Strain**		
*E. coli* TOP10	F^–^, Φ80*lac*ZΔM15, Δ*lac*X74, and *rec*A1	Invitrogen
**Plasmid**		
pET-21a	Amp^R^, T7 promoter, and lac operator	Novagen
pCadC	pET-21a derivative containing *cadC* promoter region and *cadC* cloned into *Bgl*II and *Xba*I sites	This study
pCadR	pET-21a derivative containing *cadR* and divergent *cadR* promoter region cloned into *Bgl*II and *Xba*I sites	This study
pT-GFP	T vector carrying *egfp*	Hui et al., 2018c
pT-RFP	T vector carrying *mcherry*	Hui et al., 2019
pCadC-G	pCadC derivative carrying promoterless *egfp* cloned into *Nde*I and *Hin*dIII sites	This study
pCadR-R	pCadR derivative carrying promoterless *mcherry* cloned into *Nde*I and *Hin*dIII sites	This study
pCadC-G-CadR-R	pCadC-G derivative containing the CadR-based biosensing module cloned into *Hin*dIII and *Xho*I sites	This study

*The cloning/expression regions of recombinant plasmids used in this study are shown in Supplementary Figure 1.*

### Assembly of the Genetic Constructs for Biosensing of Bioavailable Cadmium

**FIGURE 1:**
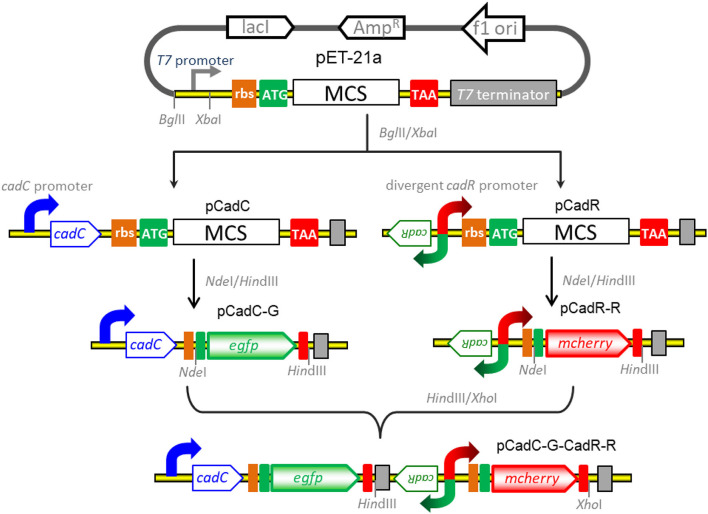
Genetic assembly and merger of two independent biosensing modules into one genetic construct for the detection of bioavailable cadmium. The green fluorescent reporter was placed downstream of the *cadC* promoter and *cadC* gene (pCadC-G). The red fluorescent reporter was placed under the regulation of the native *cadR* promoter (pCadR-R). Two independent sensing modules were integrated into one genetic construct (pCadC-G-CadR-R).

The strategy used for the construct of vectors for detection of bioavailable cadmium is shown in Figure 1. All recombinant vectors were generated using a combination of PCR and subcloning techniques as described previously (Hui et al., 2018a,b, 2020b). Two kinds of Cd(II)-sensing transcription regulators were employed to assemble cadmium biosensing systems. The DNA fragment containing the *cadC* promoter and the *cadC* gene derived from *Staphylococcus aureus* plasmid pI258 (NCBI accession no. NC_013319.1) was synthesized and inserted into the *Bgl*II-*Xba*I site of the pET-21a vector to generate pCadC. The DNA fragment containing the *cadR* gene and the divergent *cadR* promoter derived from *Pseudomonas putida* (NCBI accession no. AF071413.3) was synthesized and cloned into the pET-21a vector as described above, producing pCadR. A promoterless *egfp* gene was PCR amplified from the pT-GFP vector and subcloned into the pCadC vector using the *Nde*I and *Hin*dIII restriction sites to produce pCadC-G. A promoterless *mcherry* was PCR amplified from the pT-RFP vector and subcloned into the pCadR as described above, resulting in pCadR-R. The CadR-based bioreporter system was combined with the CadC-based bioreporter system by introducing the CadR-based bioreporter module into the *Hin*dIII and *Xho*I sites of pCadC-G, resulting in pCadC-G-CadR-R, which was designed as a double-color fluorescence biosensing system with dual-sensing modules. The DNA sequence and biological annotation of inserted fragments from the recombinant vectors used in the study are shown in Supplementary Figure 1. All the resultant constructs mentioned above were used for transformation into *E. coli* TOP10 competent cells, and the engineered whole-cell biosensors were selected on LB agar plates containing 50 μg/ml ampicillin.

**FIGURE 2:**
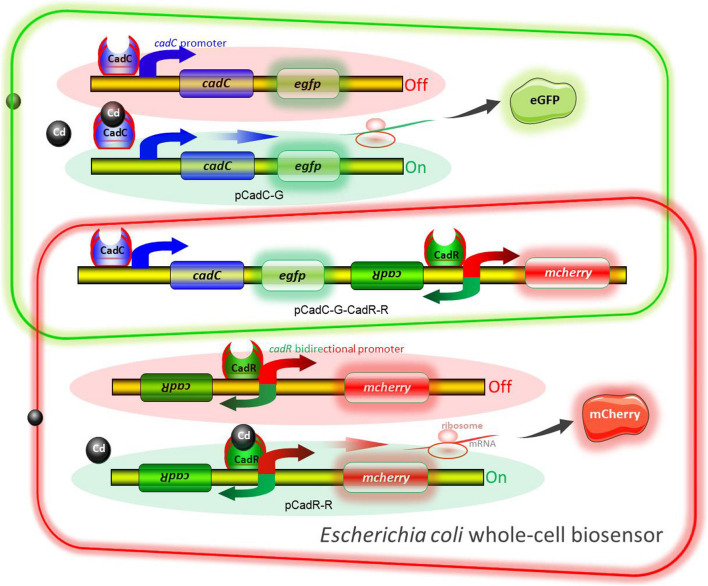
Models for single and double fluorescent indication of cadmium exposure. The apo-form dimeric CadC bound to the *cadC* promoter represses transcription of the green fluorescent reporter. The binding of Cd(II) causes the conformational change of dimeric CadC, and then, it will dissociate from the *cadC* promoter to activate the transcription of the green fluorescent reporter (schematic in green box). The dimeric CadR acts as both a transcription repressor (with no cadmium exposure) and an activator (with intracellular cadmium exposure). When the concentration of intracellular Cd(II) increases, dimeric CadR bound to the *cadR* divergent promoter will activate transcription of the red fluorescent reporter (schematic in red box). With the integration of two independent sensing modules, the resultant biosensor cell can detect Cd(II) with double-color fluorescence output (overlap between the green and red boxes).

The molecular mechanism of biodetection of Cd(II) is shown in Figure 2. The T7lac promoter located in pET-21a was substituted by Cd(II) sensory element P*cadC*-*cadC* or *cadR*-P*cadR*. A promoterless *egfp* and *mcherry* were then inserted downstream of P*cadC*-*cadC* and *cadR*-P*cadR*, respectively. The resultant single-sensing biosensors can detect target Cd(II) with green or red fluorescent signal. A dual-sensing biosensor was finally assembled by integrating two sets of Cd(II) biosensing modules into one genetic construct. Both green and red fluorescence with its independent metal-responsive property were emitted upon target Cd(II) exposure.

### Specificity Test

Single colonies from TOP10/pCadC-G, TOP10/pCadR-R, and TOP10/pCadC-G-CadR-R were picked up to inoculate 3 ml of LB medium and cultured at 37°C for 12 h. Overnight cultures were inoculated into fresh LB medium at 1% inoculation amount. Stock solutions of Cd(II), Pb(II), Zn(II), Cu(II), Mn(II), Ni(II), Ca(II), and Mg(II) were added at a final concentration of 5, 25, or 125 μM. A final concentration of 5 μM Hg(II) was added to the culture. After culturing at 37°C for 12 h, both of the fluorescent signals and bacterial density were determined. Aliquots of 100 μl induced culture were transferred into a 96-well microplate, and bacterial cell density was measured by monitoring the optical density at 600 nm using a microplate reader (BioTek Epoch, Winooski, VT, United States).

To evaluate the influence of non-responsive metal ions on the response of the dual-sensing biosensor toward Cd(II), the mixtures of 5 μM Cd(II) with other non-responsive metal ions separately or in combination at the concentration of 5 μM were supplemented into the lag phase cultures of TOP10/pCadC-G-CadR-R. After culturing at 37°C for 12 h, both of the fluorescent signals and bacterial density were determined.

To investigate the influence of responsive non-target metal ions Pb(II) or Hg(II) on the response of TOP10/pCadC-G-CadR-R toward Cd(II), the mixtures of 5 μM Cd(II) with 0, 2.5, 5, 10, and 20 μM Pb(II) or the mixtures of 5 μM Cd(II) with 0, 1.25, 2.5, 5, and 10 μM Hg were added into the lag phase cultures of TOP10/pCadC-G-CadR-R. After an overnight induction, the fluorescent intensities of double-color fluorescence and bacterial density were determined.

### Sensitivity Test

Overnight cultures of TOP10/pCadC-G, TOP10/pCadR-R, and TOP10/pCadC-G-CadR-R were inoculated into fresh LB medium at 1% inoculation amount. Double dilution method was performed as follows: the first tube was filled with 4 ml of inoculated cultures spiked with Cd(II) (the final concentration is 400 μM), and the other 15 tubes were filled with 2 ml of inoculated cultures. Two milliliters from the first tube culture was pipetted into the second tube culture. After thorough mixing, 2 ml of the second tube culture was then pipetted into the third tube culture, and so on. Finally, the induced groups with 400, 200, 100, 50, 25, 12.5, 6.25, 3.125, 1.56, 0.78, 0.39, 0.2, 0.1, 0.05, 0.025, and 0 μM Cd(II) exposure were obtained. This double dilution method has been described previously in other papers (Guo et al., 2021; Zhang et al., 2021). The resultant cultures were grown at 37°C for 12 h before measurement of fluorescent signals and bacterial cell density.

### Detection of Environment Water Samples Spiked With Cadmium

To investigate the capability of the developed dual-sensing biosensor to detect bioavailable Cd(II) in environmental water samples, overnight culture of TOP10/pCadC, TOP10/pCadR, and TOP10/pCadC-G-CadR-R was inoculated into fresh LB medium prepared with purified water, tap water from the laboratory, and lake water from a local park as described previously (Hui et al., 2020a). Lag phase cultures of TOP10/pCadC, TOP10/pCadR, and TOP10/pCadC-G-CadR-R were spiked with 0, 12.5, 25, 50, 100, and 200 μM Cd(II). After an overnight culture at 37°C, the intensities of double-color fluorescence and bacterial cell density were determined.

### Measurements of Fluorescent Signals

Both of the eGFP and mCherry fluorescent proteins generated from three whole-cell biosensors with Cd(II) exposure were quantitated with a Lumina Fluorescence Spectrometer (Thermo Fisher Scientific, Waltham, MA, United States) as previously described (Hui et al., 2019). After the cultures were properly diluted in purified H_2_O, 3 ml of sample was added to a low fluorescence background quartz cuvette. The excitation wavelength was set at 488 nm, and the intensity of emitted eGFP fluorescence was recorded at 507 nm. The excitation wavelength was set at 587 nm, and the intensity of emitted mCherry fluorescence was recorded at 610 nm. The fluorescent signal is indicated as a fluorescence count value (unit = cnt), and normalized by dividing by the OD_600_ value of the same culture (Hui et al., 2018c).

### Fluorescence Imaging of Whole-Cell Biosensors

Biosensor cell pellets were collected after centrifugation and washed twice with purified H_2_O. An aliquot of cell suspension (about 50 μl) was spread onto a clean glass slide and air-dried at room temperature. Fluorescence images were taken under a fluorescence microscope (Nikon Eclipse Ni, Tokyo, Japan) at × 400 magnification using fluorescein isothiocyanate (FITC) and Texas Red filters as described previously (Hui et al., 2018c).

## Results and Discussion

### Heavy Metal Specificity of Single-Sensing Biosensors

To adapt to environmental changes and to maintain metal homeostasis, various microorganisms coincidentally evolved a series of heavy metal detoxification systems. There are a substantial number of transcriptional regulators, such as *Cyanobacterium anabaena* AztR (Liu et al., 2005), *Oscillatoria brevis* BxmR (Liu et al., 2004), *Staphylococcus aureus* CadC (Endo and Silver, 1995), *Mycobacterium tuberculosis* CmtR (Cavet et al., 2002), *Synechococcus elongatus* SmtB (Vanzile et al., 2002), *Pseudomonas putida* CadR (Lee et al., 2001), and *E. coli* ZntR (Brocklehurst et al., 1999), identified to recognize cadmium ions. Some regulators among them, including ZntR, CadC, and CadR, have been used as the sensory elements to develop biosensors for detection of bioavailable toxic cadmium (Yoon et al., 2016b; Bereza-Malcolm et al., 2017; Kumar et al., 2017; Guo et al., 2021).

The *cadCA* operon was initially identified from plasmid pI258 of *Staphylococcus aureus*, and it was verified to confer bacterial resistance to Cd(II), Pb(II), and Zn(II) (Endo and Silver, 1995; Sun et al., 2001). An *E. coli* whole-cell biosensor based on the expression of GFP under the control of CadC could detect Cd(II) in milk samples, and the CadC-based biosensor did not respond to Pb(II) and Zn(II) both at a concentration of 100 μg/l (Kumar et al., 2017). Six *cadC* homologs were found in the genome of a marine bacterium. After the heavy metal responses of the encoded CadC-like regulators were characterized, CadC homolog-controlled T7 RNA transcription systems for biosensing signal amplification were developed, and the resultant biosensors could recognize Cd(II) and Pb(II) (Kim et al., 2016).

**FIGURE 3:**
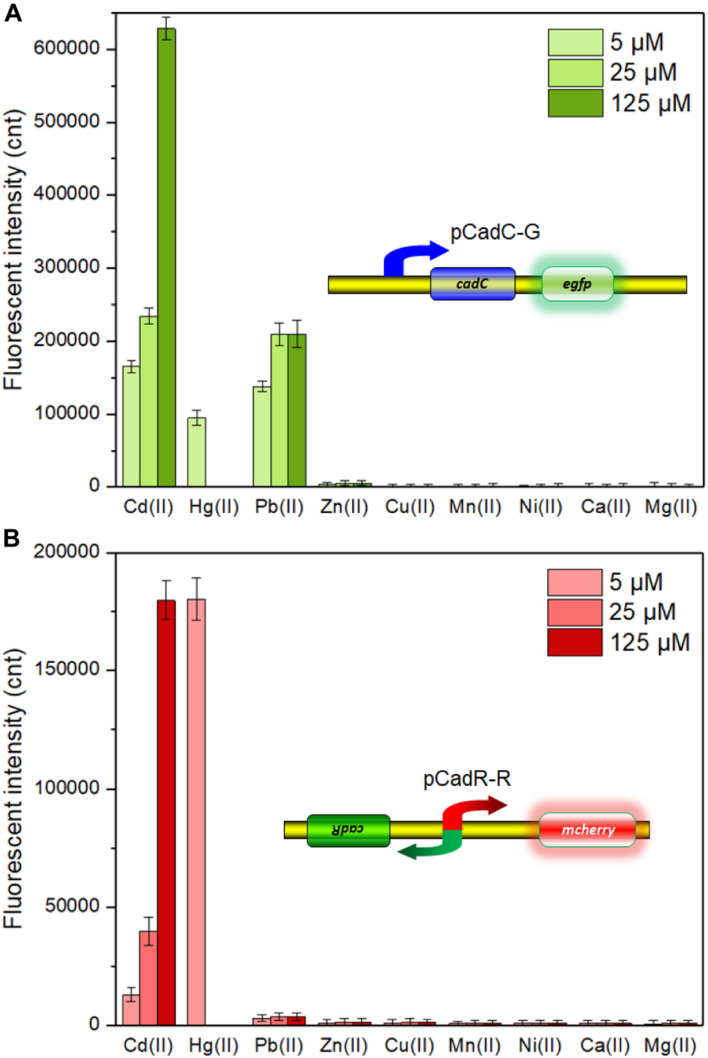
The responses of single-sensing biosensors toward nine different metal ions. After being exposed to 5, 25, and 125 μM concentrations of the specifically labeled metal ions at 37°C for 12 h, both of fluorescent signals were determined. **(A)** TOP10/pCadC-G. **(B)** TOP10/pCadR-R. The fluorescent signal was indicated as a fluorescence count value (unit = cnt), and fluorescent values were divided by the optical density at 600 nm. Results are the average of at least three independent experiments performed in triplicate. The data values shown for each metal exposure group were obtained by subtracting the control values (with no metal exposure) from the experimental values.

A natural *cadCA* operon consists of the transcriptional regulator gene *cadC* and heavy metal efflux pump gene *cadA*, which is located downstream of the *cadC* gene (Endo and Silver, 1995). An artificial *cadCA* operon was assembled in this study by substituting the *cadA* gene with the *egfp* gene. In order to enhance the expression efficiency of the reporter eGFP, an extra ribosome binding site was introduced into the upstream of the eGFP-encoding sequence (Supplementary Figure 1). To further identify the heavy metal selectivity of the CadC-based biosensor, different concentrations of Cd(II), Pb(II), Zn(II), Cu(II), Mn(II), Ni(II), Ca(II), Mg(II), and Hg(II) were exposed to TOP10/pCadC-G during the lag phase. After an overnight incubation, the green fluorescent signals were determined. As shown in Figure 3A, the whole-cell biosensor harboring pCadC-G showed strong responses toward Cd(II) and Pb(II) and a weak response toward Hg(II). The green fluorescent intensities of TOP10/pCadC-G toward Cd(II) and Pb(II) were similar at the concentrations of 5 and 25 μM, but the green florescent intensity of TOP10/pCadC-G toward Pb(II) was not further enhanced with 125 μM Pb(II) exposure. The whole-cell biosensor with CadC as the sensory element showed a significant response toward Hg(II), which was reported for the first time. To further investigate the response profile of TOP10/pCadC-G against Cd(II), Pb(II), and Hg(II), lag phase cultures of TOP10/pCadC-G were exposed to 0, 0.39, 0.78, 1.56, 3.125, 6.25, 12.5, 25, and 50 μM from three different metal ions. The green fluorescent signals were detected after a 12-h incubation at 37°C (Supplementary Figure 2A). A weak response rate increased first with less than 6.25 μM Hg(II) induction and then decreased subsequently due to the cytotoxic effect of Hg(II). Although the induction of Pb(II) exerted no significant effect on the growths of TOP10/pCadC-G, the response toward Pb(II) was not further increased with greater than 25 μM Pb(II) induction. Unlike these two non-target metal ions, Hg(II) and Pb(II), with varying responses, the response toward Cd(II) increased predictably with the concentration of Cd(II).

The *cadRA* operon was initially found in *Pseudomonas putida*, and it was demonstrated to be fully responsible for cadmium resistance and partially for zinc resistance (Lee et al., 2001; Brocklehurst et al., 2003). Crystallographic and spectroscopic studies revealed that the heavy metal specificity of CadR is mediated by the cooperation of thiolate-rich site I and histidine-rich site II (Liu et al., 2019). Previously developed whole-cell biosensors with CadR as the sensory element were verified to be induced by both Cd(II) and Hg(II) (Tao et al., 2013; Bereza-Malcolm et al., 2017; Cayron et al., 2020; Guo et al., 2021). Consistent with previous reports, the response of whole-cell biosensor TOP10/pCadR-R toward Cd(II) increased in a dose–response relationship. However, a very strong response of TOP10/pCadR-R toward 5 μM Hg(II) was found under our experimental conditions (Figure 3B). To further study the response profile of TOP10/pCadR-R against Cd(II) and Hg(II), lag phase cultures of TOP10/pCadR-R were exposed to 0, 0.39, 0.78, 1.56, 3.125, 6.25, 12.5, 25, and 50 μM from two different metal ions. The red fluorescent signals were detected after a 12-h incubation at 37°C (Supplementary Figure 2B). Compared with the Cd(II) exposure group, a significantly higher response rate was found with less than 6.25 μM Hg(II) induction. Due to the obvious cytotoxic effects of above 12.5 μM Hg(II), the red fluorescent signal decreased rapidly with above 12.5 μM Hg(II) induction. Literature studies demonstrate a similar trend of a strong response of biosensors with CadR-like regulators as sensory elements to Hg(II) (Tao et al., 2013; Cayron et al., 2020; Guo et al., 2021). Unfortunately, there are still no known or limited approaches to address this issue of the ceiling effect.

### Differential Responses of Single-Sensing Biosensors to Cadmium

Previous studies have showed that heavy metal selectivity of the MerR family is generally stronger than heavy metal selectivity of the SmtB/ArsR family (Jung and Lee, 2019). Our results above showed that the biosensor with MerR-like regulator CadR as the sensory element responded to Cd(II) and Hg(II), and the biosensor with the SmtB/ArsR family member CadC as the sensory element responded to Cd(II), Hg(II), and Pb(II). In addition, the sensitivity and strength of heavy metal response are variable among these metalloregulators (Jung and Lee, 2019). Native characteristics of a metalloregulator largely determine the overall performance of a biosensor.

**FIGURE 4:**
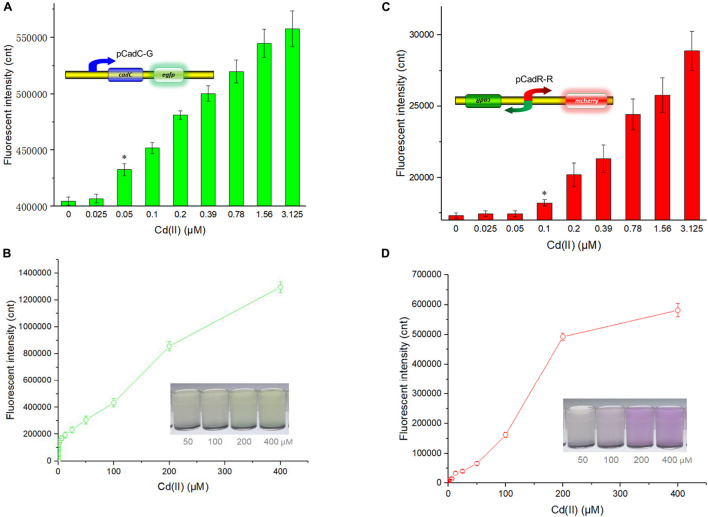
Fluorescent signals generated by single-sensing biosensors after exposure to gradient concentrations of Cd(II). After exposure to gradient concentrations of Cd(II) generated from a double dilution method, both fluorescent signals were determined after 12 h of incubation at 37°C. Fluorescence intensity values were normalized using the absorbance at 600 nm. Results are the average of at least three independent experiments performed in triplicate. Detection limit of whole-cell biosensor TOP10/pCadC-G **(A)**. The asterisk indicates a significant increase (two-tailed *t*-test, *P* < 0.001) in fluorescent intensity, in comparison to the same biosensor exposed to 0 μM Cd(II). Detection limit of whole-cell biosensor TOP10/pCadR-R **(C)**. The asterisk indicates a significant increase (two-tailed *t*-test, *P* < 0.01) in fluorescent intensity, in comparison to the same biosensor exposed to 0 μM Cd(II). Response curves of whole-cell biosensor **(B)** TOP10/pCadC-G and **(D)** TOP10/pCadR-R in response to different Cd(II) concentrations. The fluorescent signal was indicated as a fluorescence count value (unit = cnt). The data values shown for each metal exposure group were obtained by subtracting the control values (with no metal exposure) from the experimental values. The typical photographs of engineered bacterial cell cultures induced by the above 50 μM Cd(II) are shown.

Besides specificity, sensitivity is another key feature of suitable biosensors. Following analyses of the metal selectivity of two single-sensing biosensors, the detection limit was primarily determined by exposing lag phase whole-cell biosensors to a concentration gradient of Cd(II) generated using a double dilution method. Interestingly, the sensitivity of two single-sensing biosensors differed. TOP10/pCadC-G was more responsive with a significant increase in green fluorescent signal in response to 0.05 μM Cd(II) (Figure 4A). A CadC-based biosensor with GFPmut3a as the reporter was previously developed, and the detection limit was 0.041 μM (Kumar et al., 2017). TOP10/pCadR-R was responsive with a significant increase in red fluorescent signal in response to 0.1 μM Cd(II) (Figure 4C), and it was consistent with our previous data that showed that single-signal, dual-signal, and triple-signal output CadR-based biosensors responded to as low as 0.1 μM Cd(II) in supplemented M9 medium (Guo et al., 2021).

Both of the specific fluorescence changes in TOP10/pCadC-G (Figure 4B) and TOP10/pCadR-R (Figure 4D) were dramatically increased in a concentration-dependent manner. In the case of TOP10/pCadC-G, the green fluorescence was increased quickly with exposure to low concentration of Cd(II), when compared with that of TOP10/pCadR-R. In addition, TOP10/pCadC-G exhibited a nearly linear increase in green fluorescence as the concentration of Cd(II) was increased from 12.5 to 400 μM (Figure 4B). Previously developed CadC-based biosensors were demonstrated to respond over a narrow concentration range (0–30 μM), which might be the result of the differences in the genetic circuit design, the host, and the induction conditions (Kim et al., 2015, 2016; Kumar et al., 2017). No significant decrease in bacterial growth was observed with up to 400 μM Cd(II) exposure (Supplementary Figure 3). A wide responsive concentration range (0–400 μM) was observed in this study, and it will facilitate the ability to directly detect water samples heavily contaminated with cadmium.

Regardless of heavy metal cytotoxicity, many studies have shown that the saturation of metal-binding sites of MerR-like regulators would lead to constant responses in whole-cell biosensors (Hou et al., 2015; Yoon et al., 2016b; Guo et al., 2019b, 2021; Hui et al., 2020a). The rising trend of the red fluorescence in TOP10/pCadR-R obviously slowed down with above 200 μM Cd(II) exposure (Figure 4D). No continuous increase in the biosensing signals was observed in previously developed CadR-based biosensors within non-cytotoxic concentration of cadmium exposure (Tao et al., 2013; Guo et al., 2021). The saturation of metal-binding sites of low-level-background-expressed CadR may be the underlying cause, and it is very common in other developed biosensors using MerR-like regulators (Joe et al., 2012; Guo et al., 2019a; Hui et al., 2020a; Zhang et al., 2021). Furthermore, the accumulation of red fluorescent protein mCherry responsive to above 50 μM Cd(II) (Figure 4D) can be obviously distinguished by the naked eye when compared with the accumulation of green fluorescent protein eGFP (Figure 4B).

### Heavy Metal Selectivity Detection With the Dual-Sensing Biosensor

Existing metalloregulators cannot specifically discriminate cadmium from other heavy metals with similar physical and electrochemical characteristics (Tao et al., 2013; Cayron et al., 2019; Jung and Lee, 2019; Guo et al., 2021). With the accumulation of biochemical data of diverse cadmium transcriptional regulators, integration of multiple cadmium sensory elements into one biosensing system is novel and instrumental in the development of biosensors.

**FIGURE 5:**
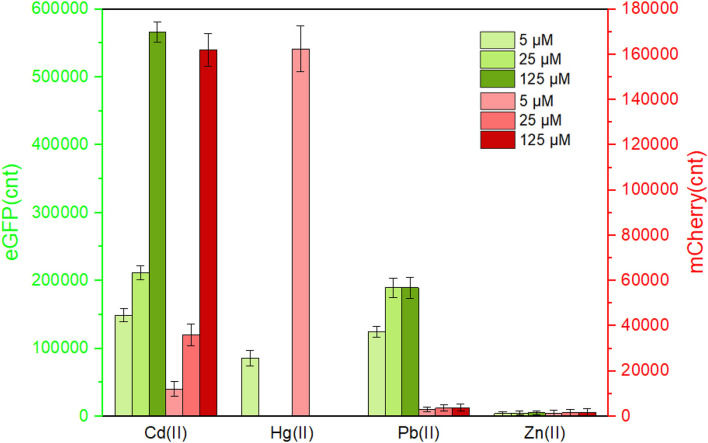
The response of the dual-sensing biosensor toward different metal ions. Double-color fluorescence of whole-cell biosensor TOP10/pCadC-G-CadR-R in the presence of different metal ions was determined after a 12-h incubation at 37°C. Fluorescent values were divided by the optical density at 600 nm. The data values shown for each metal exposure group were obtained by subtracting the control values (with no metal exposure) from the experimental values. Results are the average of at least three independent experiments performed in triplicate.

Following the characterization of CadC-based and CadR-based single-sensing biosensors, two sets of Cd(II) biosensing module spacing with a *rrn*B transcription terminator were assembled in one genetic construct. After transformation into the host TOP10, the resultant whole-cell biosensor TOP10/pCadC-G-CadR-R was exposed to various concentrations of Cd(II), Hg(II), Pb(II), and Zn(II). After an overnight incubation, the green fluorescence generated from the CadC-eGFP system and the red fluorescence generated from the CadR-mCherry system were simultaneously detected (Figure 5). Rational gene circuit design allows integrating multiple sensing elements on the same biological device, and their respective response properties can be well retained (Yoon et al., 2016b; McNerney and Styczynski, 2017; McNerney et al., 2019). Two sets of Cd(II)-sensing modules are connected in series on one genetic construct in this study. As expected, a combination of two sets of Cd(II) biosensing systems did not change their respective metal selectivity. Consistent with the above results, the green fluorescence responding to Cd(II), Hg(II), and Pb(II) was detected, and the red fluorescence responding to Cd(II) and Hg(II) was detected. The intensity of fluorescent signaling from a dual-sensing biosensor is slightly decreased when compared to the intensity of fluorescent signaling from its respective single-sensing biosensor.

To further characterize the response profile of the dual-sensing biosensor against Cd(II), Pb(II), and Hg(II), lag phase cultures of TOP10/pCadC-G-CadR-R were exposed to 0–50 μM of three different metal ions. The dual-color fluorescent signals were detected after a 12-h incubation at 37°C (Supplementary Figure 4). The capability of this dual-sensing biosensor was demonstrated to maintain similar response profiles of respective signal-sensing biosensors. No significant red fluorescence toward Pb(II) was detected, and the green fluorescence toward Pb(II) was gradually increased to its maximum value within 25 μM Pb(II) induction. Both green and red fluorescent signals toward Hg(II) were significantly decreased with greater than 6.25 μM Hg(II) induction due to the cytotoxic effect of Hg(II). However, the dual-color fluorescent signals toward Cd(II) has been proportionally increasing with the concentration of Cd(II).

### Performance of the Dual-Sensing Biosensor With Cadmium and Interfering Metal Ion Exposure

**FIGURE 6:**
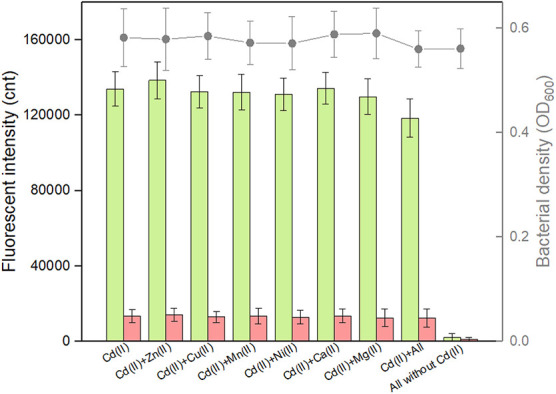
Influence of non-responsive metal ions on the response of the dual-sensing biosensor toward Cd(II). Double-color fluorescence derived from TOP10/pCadC-G-CadR-R exposed with 5 μM Cd(II) in the presence of various non-responsive metal ions. After a 12-h incubation at 37°C, bacterial cell density (OD_600_) was measured (point line diagram, right-Y scale) using a microplate reader, and both eGFP (green bars) and mCherry (red bars) fluorescent intensities were determined using a fluorescence spectrophotometer (bar chart, left-Y scale). The fluorescent signal was indicated as a fluorescence count value (unit = cnt), and fluorescence intensity values were normalized using the absorbance at 600 nm. The data values shown for each metal exposure group were obtained by subtracting the control values (with no metal exposure) from the experimental values. Results are the average of at least three independent experiments performed in triplicate.

Complex pollutants including various metal ions coexist in contaminated environmental water. A good anti-jamming capability is important for engineered whole-cell biosensor to assess Cd(II)-contaminated environmental water. The above results demonstrated that both CadC-based and CadR-based biosensors responded silently to the vast majority of metal ions such as Zn(II), Cu(II), Mn(II), Ni(II), Ca(II), and Mg(II). The influence of these non-responsive metal ions on the response of the dual-sensing biosensor toward target Cd(II) was further investigated. As shown in Figure 6, all these non-responsive metal ions in combination resulted in the response of double-color fluorescence that was similar with the basal expression. Importantly, double-color fluorescence toward 5 μM Cd(II) was not significantly influenced by these non-responsive metal ions. In the presence of non-responsive metal ions alone, TOP10/pCadC-G-CadR-R still emitted double-color fluorescence that was comparable to the double fluorescent signals with Cd(II) exposure alone (Figure 6).

**FIGURE 7:**
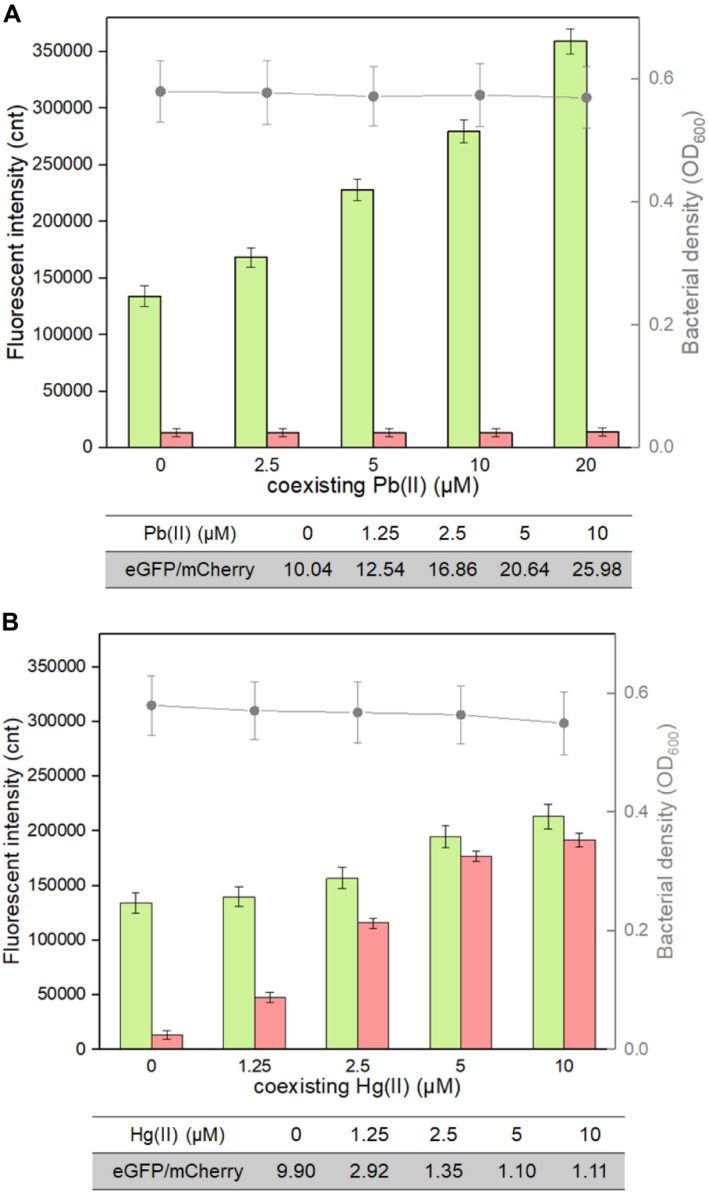
Influence of responsive non-target Pb(II) and Hg(II) on the response of the dual-sensing biosensor toward Cd(II). Double-color fluorescence derived from TOP10/pCadC-G-CadR-R exposed to 5 μM Cd(II) in the presence of increased concentrations of **(A)** Pb(II) and **(B)** Hg(II). After a 12-h incubation at 37°C, bacterial cell density was measured (point line diagram, right-Y scale), and both eGFP (green bars) and mCherry (red bars) fluorescence were determined (bar chart, left-Y scale). The fluorescence intensity ratios (eGFP/mCherry) were shown in the tables below the corresponding figures. The fluorescent signal was indicated as a fluorescence count value (unit = cnt), and fluorescence intensity values were normalized using the absorbance at 600 nm. The data values shown for each metal exposure group were obtained by subtracting the control values (with no metal exposure) from the experimental values. Results are the average of at least three independent experiments performed in triplicate.

The weakness of cadmium single-sensing biosensor can be partly overcome by a combination of two biosensing systems. For example, the dual-sensing biosensor will conveniently recognize Pb(II) exposure alone when only green fluorescence is emitted (Figure 5). To further evaluate the interference of responsive non-target metal ions on the performance of the dual-sensing biosensor, TOP10/pCadC-G-CadR-R was induced with 5 μM Cd(II) alone or in combination with elevated concentrations of Pb(II) or Hg(II). Until the double-color fluorescent signals were stable, the fluorescent intensities and bacterial cell densities were determined (Figure 7). Only the rise in green fluorescence generated from CadC-eGFP biosensing system was accompanied with elevated concentrations of Pb(II), and it led to the significantly enhanced ratios of eGFP/mCherry owing to the stable red fluorescence (Figure 7A). The situation was different when TOP10/pCadC-G-CadR-R was treated with Cd(II) and Hg(II). Both CadC-eGFP and CadR-mCherry biosensing systems responded to Hg(II). However, a significantly stronger mCherry fluorescence was generated from CadR-mCherry biosensing system (Figure 5). Due to the strong response of CadR-mCherry biosensing system toward non-target Hg(II), incorporation of Hg(II) into the culture leads to bigger rise in red fluorescence than rise in green fluorescence, and it was conveniently judged by the decreased ratios of eGFP/mCherry (Figure 7B). Although the combination of two single-sensing systems could not enhance the metal selectivity of whole-cell biosensor, the findings above showed that the dual-sensing biosensor was effective in distinguishing non-target-responsive metal ions from target metal ions.

The weak specificity of the developed Cd(II) whole-cell biosensors was verified to be largely determined by the intrinsic properties of available metalloregulators. Novel biosensors specific for Cd(II) are expected to be developed using newly discovered Cd(II)-responsive metalloregulators. Cadmium-specific mutants of available metal-sensing regulators have been shown to improve cadmium specificity (Hakkila et al., 2011; Tao et al., 2013). In addition, the genetic logic gate was shown to function as a biological filter and amplifier to enhance the selectivity and sensitivity of whole-cell biosensors (Wang et al., 2013). Our findings suggest that the development of dual-sensing biosensor is another alternative to improve the performance of biosensors. A number of studies have demonstrated that metalloregulator MerR responded selectively to Hg(II). MerR-based whole-cell biosensors all showed extraordinary selectivity toward Hg(II) (Hansen and Sorensen, 2000; Wang et al., 2020; Zhang et al., 2021). If CadR-based biosensing system and MerR-based biosensing system are integrated into one genetic construct, the resultant dual-sensing biosensor will be able to distinguish Cd(II) and Hg(II) simultaneously.

### Cadmium Detection With the Dual-Sensing Biosensor

**FIGURE 8:**
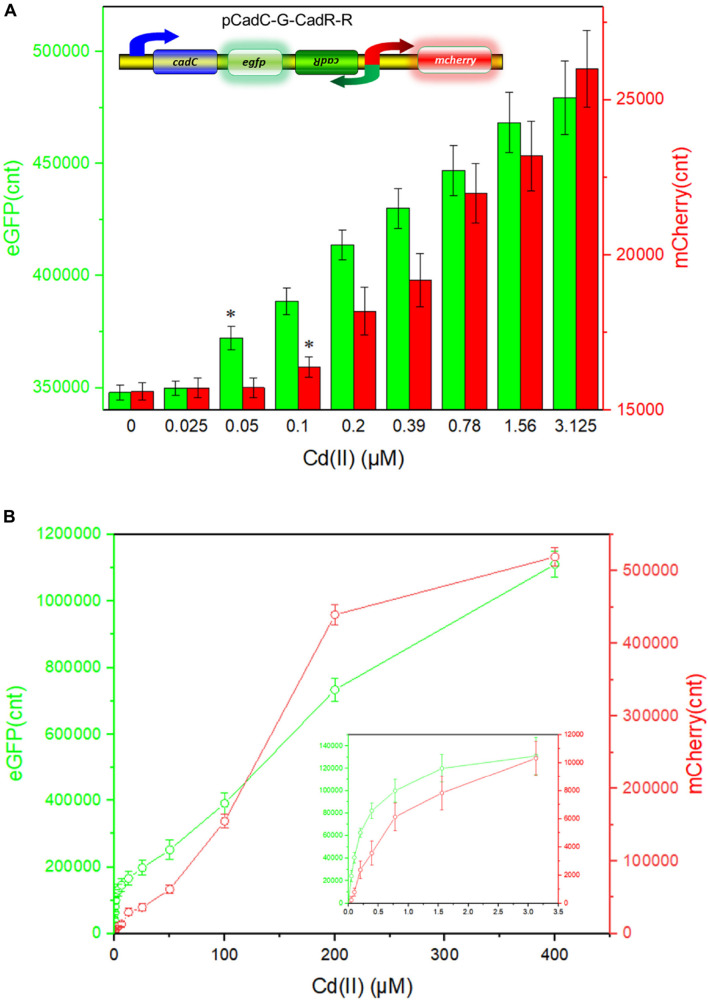
Double-color fluorescence generated by the dual-sensing biosensor after exposure to gradient concentrations of Cd(II). Both fluorescent signals were determined after a 12-h incubation with gradient concentrations of Cd(II) at 37°C. The fluorescent signal was indicated as a fluorescence count value (unit = cnt), and fluorescence intensity values were normalized using the absorbance at 600 nm. Results are the average of at least three independent experiments performed in triplicate. **(A)** Detection limit of the dual-sensing biosensor TOP10/pCadC-G-CadR-R. The asterisk indicates a significant increase (two-tailed *t*-test, *P* < 0.001 for eGFP and *P* < 0.05 for mCherry) in fluorescent intensity, in comparison to the same biosensor exposed to 0 μM Cd(II). **(B)** Response curves of the dual-sensing biosensor TOP10/pCadC-G-CadR-R in response to different Cd(II) concentrations. The data values shown for each metal exposure group were obtained by subtracting the control values (with no metal exposure) from the experimental values.

The detection limit of the dual-sensing biosensor was first determined by exposing lag phase TOP10/pCadC-G-CadR-R to a concentration gradient of Cd(II) generated using a double dilution method. It was noted that the detection sensitivity of the combined dual-sensing system is consistent with that of the independent single-sensing system. The CadC-eGFP system was found to respond to the lowest concentration of Cd(II) at 0.05 μM with green fluorescence, and the CadR-mCherry system could detect Cd(II) at a concentration of 0.1 μM with red fluorescence (Figure 8A).

To investigate the response characteristics of the dual-sensing biosensor toward Cd(II), double-color fluorescence responsive to Cd(II) ranging from 0 to 400 μM was then determined. As shown in Figure 8B, both of the fluorescent signals toward Cd(II) increased in a dose–response relationship with increasing Cd(II) exposure concentrations. More importantly, no obvious interference effect was observed when two sets of biosensing system were integrated into one genetic construct. Both response properties of the two biosensing modules were still unchanged in the dual-sensing biosensor. Compared with a single-sensing biosensor, the production of two reporters may increase energy and nutrient consumption, and the capture of two dimeric metalloregulatory proteins may also contribute to decreased concentration of intracellular available Cd(II). Thus, slightly attenuated fluorescent signal strength is expected in the dual-sensing biosensor. Nonetheless, this is the first report of the development of a novel dual-sensing whole-cell biosensor for simultaneous detection of bioavailable cadmium. The simultaneous application of two biosensing modules provides versatile biosensing signals and improved performance.

Time–response curves of TOP10/pCadR-R, TOP10/pCadC-G, and TOP10/pCadC-G-CadR-R toward 100 μM Hg(II) are shown in Supplementary Figure 5. It was surprising that the response characteristics of two Cd(II) bioreporter systems were nearly consistent. Fluorescent signals from either two single-sensing biosensors or the dual-sensing biosensor were all increased with the extension of induction time. Fluorescent signals generated from both CadR-based and CadC-based bioreporter systems were not significantly increased after an 8-h induction. In addition, the background responses of two Cd(II) bioreporter systems without Cd(II) exposure were also tested. The basal expression characteristics were also similar with Cd(II)-induced expression characteristics, and the basal expression levels of two fluorescence proteins were not enhanced after 8-h incubation (data not shown). An overnight induction was chosen in the current study to facilitate the experimental arrangement. Interestingly, an 8-h induction time was actually enough to obtain the maximum fluorescence output.

### Performance of the Dual-Sensing Biosensor Treated With Environmental Water Samples

**FIGURE 9:**
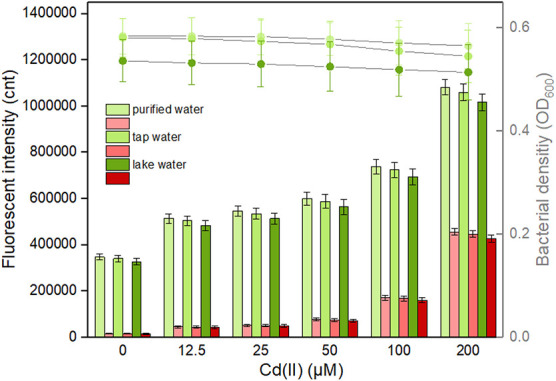
Responses of the dual-sensing biosensor cell toward environmental water samples spiked with different concentrations of Cd(II). Luria-Bertani (LB)-incubated lag-phase cultures of the dual-sensing biosensor TOP10/pCadC-G-CadR-R were exposed to varying concentrations of Cd(II) in the following water samples: purified water, tap water, and lake water. After a 12-h incubation at 37°C, bacterial cell density was measured (point line diagram, right-Y scale), and both eGFP (green bars) and mCherry (red bars) fluorescence were determined (bar chart, left-Y scale). The fluorescent signal was indicated as a fluorescence count value (unit = cnt), and fluorescence intensity values were normalized using the absorbance at 600 nm. The green fluorescent intensity values of TOP10/pCadC-G-CadR-R were obtained by subtracting that of TOP10/pCadC, and the red fluorescent intensity values of TOP10/pCadC-G-CadR-R were obtained by subtracting that of TOP10/pCadR. Results are the average of at least three independent experiments performed in triplicate.

It is well known that abundant microorganisms, organic pollutants, and inorganic pollutants exist in the natural water system. To validate the capability of bacterial dual-sensing biosensor to detect bioavailable Cd(II) in water samples from different sources, engineered TOP10/pCadC-G-CadR-R in the lag phase were exposed to 0, 12.5, 25, 50, 100, and 200 μM Cd(II) dispersed in LB culture prepared using purified water, tap water, and lake water. After an overnight induction, it was noted that an increase in the double-color fluorescence correlated with an increase in Cd(II) concentration (Figure 9). No significant decrease in both bacterial densities and fluorescent intensities was observed among the purified water-treated group and the tap water-treated group. Due to the influence of complex chemical compositions in lake water, biosensor cell density was decreased in the lake water-treated group, and the intensities of double-color fluorescence were also slightly decreased in that group. However, the increasing trend of fluorescence intensity with exposure concentration of spiked Cd(II) was still well maintained. The results of our study demonstrate that the developed dual-sensing biosensor has the ability to resist the interferences from the natural water environment, and bioavailable toxic cadmium spiked in the lake water can be preliminarily evaluated by detecting double-color fluorescence, especially when there are high concentrations of existing bioavailable cadmium.

The leakage expression is inherent and very common in microorganisms because it allows them to survive during sudden and drastic changes in the surroundings (Randall et al., 2011). Heavy metal biosensors may be triggered by targets with similar chemical properties (Amaro et al., 2011; Wang et al., 2013). Natural Cd(II) sensing elements were used in this study to develop the biosensors. As shown in Figure 9, the basal expression levels of the dual-sensing biosensor were relatively high. Strategies that act on the transcriptional process, the translational process, and the post-translational process have been demonstrated to effectively reduce leakiness (Wan et al., 2019a). The United States Environmental Protection Agency (USEPA) has recommended the criterion maximum concentration (CMC) in fresh water to be 0.027 μM for cadmium, 0.07 μM for mercury, and 0.396 μM for lead (USEPA, 1984) in which aquatic life can tolerate. The biosensors developed in this study can hardly detect below the CMC. The optimization of sensory receptor densities (Wang et al., 2015), the application of multi-layered transcriptional amplifiers (Wang et al., 2014), and the coupling by leakage regulation approaches were all established to facilitate in the development of ultrasensitive cellular biosensors for heavy metal detection (Wan et al., 2019b).

### Fluorescence Microscopic Analysis of Differential Responses of Whole-Cell Biosensors

**FIGURE 10:**
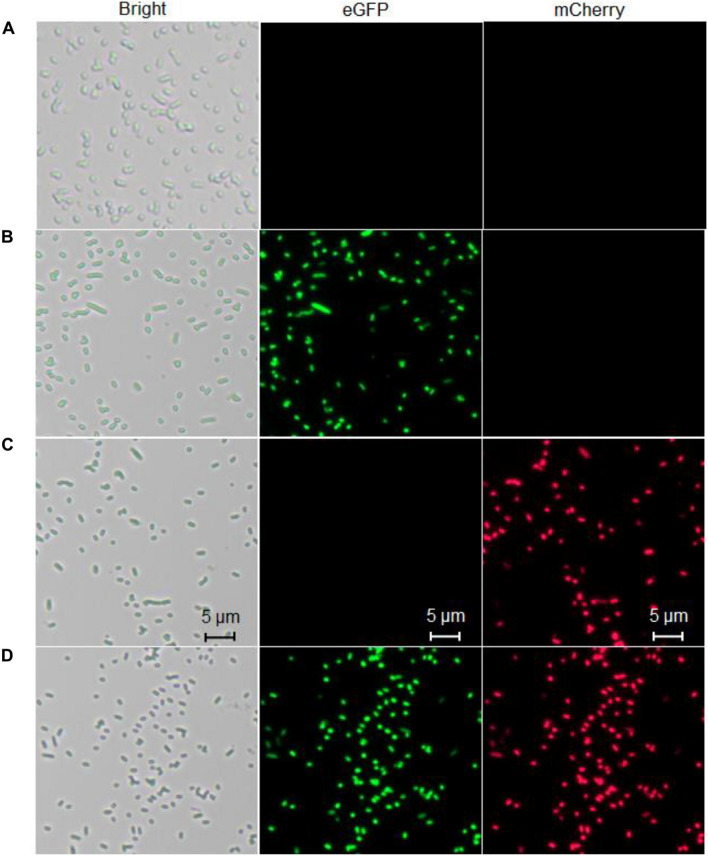
Fluorescence images of three whole-cell biosensors. **(A)** The control group, TOP10 harboring the plasmid pCadC-G-CadR-R with no Cd(II) exposure. **(B)** TOP10 harboring the plasmid pCadC-G with 100 μM Cd(II) exposure. **(C)** TOP10 harboring the plasmid pCadR-R with 100 μM Cd(II) exposure. **(D)** TOP10 harboring the plasmid pCadC-G-CadR-R with 100 μM Cd(II) exposure. After a 6-h incubation at 37°C, engineered bacterial cells harboring various biosensor vectors were observed under a fluorescence microscope (×400 magnification). The green fluorescent signal was first detected with a fluorescein isothiocyanate (FITC) filter and then the red fluorescent signal was detected using a Texas Red filter.

To confirm the differential bioindication of three whole-cell biosensors induced by their cognate metal ion Cd(II), lag phase cultures of TOP10/pCadC-G, TOP10/pCadR-R, and TOP10/pCadC-G-CadR-R were induced with 100 μM Cd(II) at 37°C for 6 h. TOP10/pCadC-G-CadR-R with no Cd(II) exposure was used as the control group. Engineered bacterial cells were spread onto the slides, and the bright field and fluorescent field images were captured and shown in Figure 10. A long-term culture of whole-cell biosensors with native metalloregulators as the sensory elements usually led to leakage expression of reporter (Yoon et al., 2016b; Guo et al., 2021). To reduce the leakage accumulation of fluorescent reporters, a 6-h incubation was adopted for the fluorescent image acquisition. No obvious fluorescent signals were detected from TOP10/pCadC-G-CadR-R with no Cd(II) exposure (Figure 10A). The green fluorescent signal was detected from TOP10/pCadC-G induced with 100 μM Cd(II), and the red fluorescent signal was detected from TOP10/pCadR-R induced with 100 μM Cd(II). Both of the fluorescent signals were detected from TOP10/pCadC-G-CadR-R treated with 100 μM Cd(II). Simultaneous differential bioindication was finally corroborated by the fluorescent microscopic analysis. Double-color fluorescent responses can also facilitate the development of flow cytometry (Silva-Rocha and De Lorenzo, 2012) and microfluidic platform (Kim et al., 2015, 2016) for reporter signal monitor.

## Conclusion

Specific recognition of target metal is the minimum requirement for the whole-cell biosensor. However, the known native existing metalloregulators currently cannot specifically respond to cadmium. This study demonstrates that the combination of multiple sensing modules led to the development of a versatile whole-cell biosensor to specifically detect bioavailable cadmium. The single-sensing CadC-eGFP system was responsive to Cd(II), Hg(II), and Pb(II). The single-sensing CadR-mCherry system was responsive to Cd(II) and Hg(II). A combination of the two single-sensing systems was shown to give two biosensing signals and facilitate in distinguishing Cd(II) from non-target-responsive metal ions Pb(II) or Hg(II). Moreover, the cadmium-responsive double-color fluorescence increased in a dose–response relationship within a certain range of cadmium concentration, and the dual-sensing biosensor showed a certain anti-jamming capability to both non-responsive metal ions in the culture and complex components in environmental water, thereby having a potential to be a quantitative biosensor for the determination of bioavailable cadmium. Our findings illustrate the importance of the combined use of multiple sensory elements for the improved performance of whole-cell biosensor devices.

## Data Availability Statement

The original contributions presented in the study are included in the article/Supplementary Material, further inquiries can be directed to the corresponding author/s.

## Author Contributions

CH designed the experimental protocol and drafted the manuscript. YG, JW, XY, and XG carried out the majority of the study. LL, YX, and JY analyzed the data. All authors read and approved the submitted version.

## Conflict of Interest

The authors declare that the research was conducted in the absence of any commercial or financial relationships that could be construed as a potential conflict of interest.

## Publisher’s Note

All claims expressed in this article are solely those of the authors and do not necessarily represent those of their affiliated organizations, or those of the publisher, the editors and the reviewers. Any product that may be evaluated in this article, or claim that may be made by its manufacturer, is not guaranteed or endorsed by the publisher.
