# Supplementary material for: Detection of Bioavailable Cadmium by Double-Color Fluorescence Based on a Dual-Sensing Bioreporter System
Source: Front Microbiol. 2021 Sep 16;12:696195. doi: 10.3389/fmicb.2021.696195 (PMC8481780; doi:10.3389/fmicb.2021.696195)
Supplement: Supplementary file 1 [file Data_Sheet_1.docx]

**AGATCT**GTGTATTTTTTAATAAATTATTTTACTTATTGAAATGTATTATTTTCTAATGTCATACCCTGGTCAAAACCGTTCGTTTTTGAGACTAGAATTTTATGCC

*cadC* promoter region

CTACTTACTTCTTTTATTTTCATTCAAATATTTGCTTGCATGATGAGTCGAAAATGGTTATAATACACTCAAATAAATATTTGAATGAAGATGGGATGATAATATGAAAAAGAAAGATACTTGTGAAATTTTTTGTTATGACGAAGAAAAGGTTAATCGAATACAAGGGGATTTACAAACAGTTGATATTTCTGGTGTTAGCCAAATTTT

*cadC*

AAAGGCTATTGCCGATGAAAATAGAGCAAAAATTACTTACGCTCTGTGTCAGGATGAAGAGTTGTGTGTTTGTGATATAGCAAATATCTTAGGTGTTACGATAGCAAATGCATCTCATCATTTACGTACGCTTTATAAGCAAGGGGTGGTCAACTTTAGAAAAGAAGGAAAACTAGCTTTATATTCTTTAGGTGATGAACATATCAGGCAGATAATGATGATCGCCCTAGCACATAAGAAAGAAGTGAAGGTCAAT

rbs

GTCTAA**TCTAGA**AATAATTTTGTTTAACTTTAAGAAGGAGATATACATATG

Multiple cloning sites

GCTAGCATGACTGGTGGACAGCAAATGGGTCGCGGATCCGAATTCGAGCTCCGTCGAC**AAGCTT**GCGGCCGCACTCGAGCACCACCACCACCACCACTGAGATCCGGCTGCTAACAAAGCCCGAAAGGAAGCTGAGTTGGCTGCT

T7 terminator

GCCACCGCTGAGCAATAACTAGCATAACCCCTTGGGGCCTCTAAACGGGTCTTGAGGGGTTTTTTG

**pCadC**

**AGATCT**TTAATGCCCGTGGCTTCGCCCTACATGCGAATGCTCGGTTTCCGGCACCGATACCGCCCCGTTCGTCTCCAGTTGCTGCAAGATCGCACACTCCGCCCCTTGTGCATTGCAGCGCCGCCGCAGCTCCACCAGCTGTTCCTGCAACGCCACCAGACCATCGATCCGTGCCTGCACATGCTCGATATGCTC

*cadR*

GTCGATCAGCGCATTGACGCTGCCGCACGAATCATCGGGGCTGTCGCGCAGGCGTAGCAGGCTGCGGATTTCATCCAGGGTCATGTCCAGGGTGCGGCAGTTGCGGATGAAGGTAAGCCGCTCGACGTGGGCCTGGGTGTACAGCCGGTAGTTGCCGTCGCTGCGTGCCGGCTCCGGCAGCAGCTGTTCACGCTCGTAGTAGCGGATGGTTTCCACGGCGCAGTCGGTGGCTTTGGCCAGTTCTCCGATCTTCATCACGAAATTCTCCAGCAAGTGGCTTGACCCTATAG

Divergent *cadR* promoter region

TGGCTACAGGGTGTTCACTTGGCAACAGGCTCAAATTAAGGATGACCCC

rbs

**TCTAGA**AATAATTTTGTTTAACTTTAAGAAGGAGATATACATATGGCTAGC

Multiple cloning sites

ATGACTGGTGGACAGCAAATGGGTCGCGGATCCGAATTCGAGCTCCGTCGAC**AAGCTT**GCGGCCGCACTCGAGCACCACCACCACCACCACTGAGATCCGGCTGCTAACAAAGCCCGAAAGGAAGCTGAGTTGGCTGCTGCCACC

T7 terminator

GCTGAGCAATAACTAGCATAACCCCTTGGGGCCTCTAAACGGGTCTTGAGGGGTTTTTTG

**pCadR**

**AGATCT**GTGTATTTTTTAATAAATTATTTTACTTATTGAAATGTATTATTTTCTAATGTCATACCCTGGTCAAAACCGTTCGTTTTTGAGACTAGAATTTTATGCC

*cadC* promoter region

CTACTTACTTCTTTTATTTTCATTCAAATATTTGCTTGCATGATGAGTCGAAAATGGTTATAATACACTCAAATAAATATTTGAATGAAGATGGGATGATAATATGAAAAAGAAAGATACTTGTGAAATTTTTTGTTATGACGAAGAAAAGGTTAATCGAATACAAGGGGATTTACAAACAGTTGATATTTCTGGTGTTAGCCAAATTTT

*cadC*

AAAGGCTATTGCCGATGAAAATAGAGCAAAAATTACTTACGCTCTGTGTCAGGATGAAGAGTTGTGTGTTTGTGATATAGCAAATATCTTAGGTGTTACGATAGCAAATGCATCTCATCATTTACGTACGCTTTATAAGCAAGGGGTGGTCAACTTTAGAAAAGAAGGAAAACTAGCTTTATATTCTTTAGGTGATGAACATATCAGGCAGATAATGATGATCGCCCTAGCACATAAGAAAGAAGTGAAGGTCAAT

rbs

GTCTAA**TCTAGA**AATAATTTTGTTTAACTTTAAGAAGGAGATATACATATGGTTTCTAAAGGCGAAGAACTGTTCACCGGCGTTGTTCCGATCCTGGTTGAACTGGATGGTGATGTTAACGGCCACAAATTCAGCGTCAGCGGCGAAGGCGAAGGCGATGCGACCTACGGCAAACTGACCCTGAAATTCATCTGCACCACCGGTAAACTGCCGGTTCCGTGGCCGACCCTGGTTACCACCCTGACCTACGGCGTTCAGTGCTTCAGCCGTTACCCGGATCACATGAAACAGCACGATTTCTTCAAAAGCGCGATGCCGGAAGGCTACGTTCAGGAACGTACCATCTTC

*egfp*

TTCAAGGATGATGGCAACTACAAAACCCGTGCGGAAGTTAAATTCGAAGGCGATACGCTGGTTAACCGTATCGAACTGAAAGGCATCGATTTCAAAGAAGATGGTAACATCCTGGGGCACAAACTGGAATACAACTACAACAGCCACAACGTTTATATCATGGCCGACAAACAGAAAAACGGAATCAAAGTTAATTTCAAGATTCGCCACAATATCGAAGACGGTTCTGTGCAACTTGCAGATCATTACCAGCAAAACACCCCAATTGGCGATGGACCCGTCCTGCTGCCGGACAACCATTACCTGTCGACACAGTCAGCGCTGTCCAAGGATCCGAACGAAAAACGTGATCACATGGTTCTGCTGGAATTCGTTACCGCGGCGGGCATCACCCTGGGTATGGATGAACTGTACAAATAA**AAGCTT**GCGGCCGCACTCGAGCACCACCACCACCACCACTGAGATCCGGCTGCTAACAAAGCCCGAAAGGAAGCTGAGTTGGCTGCTGCCACCGCTGAGCAATAACTAGCATAACCCCTTGGGGCCTCTAAACGGGTCTTGAGGGGTTTTTTG

**pCadC-G**

**AGATCT**TTAATGCCCGTGGCTTCGCCCTACATGCGAATGCTCGGTTTCCGGCACCGATACCGCCCCGTTCGTCTCCAGTTGCTGCAAGATCGCACACTCCGCCCCTTGTGCATTGCAGCGCCGCCGCAGCTCCACCAGCTGTTCCTGCAACGCCACCAGACCATCGATCCGTGCCTGCACATGCTCGATATGCTC

*cadR*

GTCGATCAGCGCATTGACGCTGCCGCACGAATCATCGGGGCTGTCGCGCAGGCGTAGCAGGCTGCGGATTTCATCCAGGGTCATGTCCAGGGTGCGGCAGTTGCGGATGAAGGTAAGCCGCTCGACGTGGGCCTGGGTGTACAGCCGGTAGTTGCCGTCGCTGCGTGCCGGCTCCGGCAGCAGCTGTTCACGCTCGTAGTAGCGGATGGTTTCCACGGCGCAGTCGGTGGCTTTGGCCAGTTCTCCGATCTTCATCACGAAATTCTCCAGCAAGTGGCTTGACCCTATAG

Divergent *cadR* promoter region

TGGCTACAGGGTGTTCACTTGGCAACAGGCTCAAATTAAGGATGACCCC

rbs

**TCTAGA**AATAATTTTGTTTAACTTTAAGAAGGAGATATACATATGGTCTCTAAAGGCGAGGAAGACAACATGGCAATCATCAAAGAGTTCATGCGTTTCAAAGTGCACATGGAGGGTAGCGTCAACGGTCACGAATTTGAAATCGAAGGTGAGGGTGAAGGTCGCCCGTACGAAGGTACCCAAACCGCTAAACTGAAAGTGACGAAAGGTGGTCCGCTGCCATTCGCATGGGATATCCTGTCTCCACAGTTCATGTACGGTTCTAAAGCGTACGTGAAACACCCGGCTGACATTCCTGACTACCTGAAACTGTCCTTCCCGGAAGGTTTCAAATGGGAACGTGTGATG

*mcherry*

AACTTCGAGGACGGTGGCGTAGTTACTGTTACCCAGGACTCTTCCCTGCAGGATGGTGAGTTTATCTACAAGGTTAAACTGCGTGGCACTAACTTTCCGTCCGACGGCCCGGTTATGCAGAAGAAGACTATGGGCTGGGAAGCATCTAGCGAACGTATGTATCCGGAAGATGGTGCTCTGAAAGGCGAAATCAAACAGCGTCTGAAACTGAAAGACGGCGGCCATTATGATGCGGAAGTTAAGACGACCTACAAAGCCAAGAAACCGGTTCAGCTGCCGGGCGCCTATAATGTAAACATCAAACTGGATATTACCTCCCACAACGAAGATTACACCATTGTAGAACAATATGAACGCGCGGAAGGCCGCCATAGCACCGGCGGCATGGACGAACTGTACAAATAA**AAGCTT**GCGGCCGCACTCGAGCACCACCACCACCACCACTGAGATCCGGCTGCTAACAAAGCCCGAAAGGAAGCTGAGTTGGCTGCTGCCACCGCTGAGCAATAACTAGCATAACCCCTTGGGGCCTCTAAACGGGTCTTGAGGGGTTTTTTG

**pCadR-R**

**AGATCT**GTGTATTTTTTAATAAATTATTTTACTTATTGAAATGTATTATTTTCTAATGTCATACCCTGGTCAAAACCGTTCGTTTTTGAGACTAGAATTTTATGCC

*cadC* promoter region

CTACTTACTTCTTTTATTTTCATTCAAATATTTGCTTGCATGATGAGTCGAAAATGGTTATAATACACTCAAATAAATATTTGAATGAAGATGGGATGATAATATGAAAAAGAAAGATACTTGTGAAATTTTTTGTTATGACGAAGAAAAGGTTAATCGAATACAAGGGGATTTACAAACAGTTGATATTTCTGGTGTTAGCCAAATTTT

*cadC*

AAAGGCTATTGCCGATGAAAATAGAGCAAAAATTACTTACGCTCTGTGTCAGGATGAAGAGTTGTGTGTTTGTGATATAGCAAATATCTTAGGTGTTACGATAGCAAATGCATCTCATCATTTACGTACGCTTTATAAGCAAGGGGTGGTCAACTTTAGAAAAGAAGGAAAACTAGCTTTATATTCTTTAGGTGATGAACATATCAGGCAGATAATGATGATCGCCCTAGCACATAAGAAAGAAGTGAAGGTCAATGTCTAA**TCTAGA**AATAATTTTGTTTAACTTTAAGAAGGAGATATACATATG--*egfp*—TAA**AAGCTT**TGCCTGGCGGCAGTAGCGCGGTGGTCCCACCTGACCCCATGCCGAACTCAGAAGTGAAACGCCGTAGCGCCGATGGTAGTGTG

*rrn*B transcription termination region

GGGTCTCCCCATGCGAGAGTAGGGAACTGCCAGGCATCAAATAAAACGAAAGGCTCAGTCGAAAGACTGGGCCTTTTAATGCCCGTGGCTTCGCCCTACATGCGAATGCTCGGTTTCCGGCACCGATACCGCCCCGTTCGTCTCCAGTTGCTGCAAGATCGCACACTCCGCCCCTTGTGCATTGCAGCGCCGCCGCAGCTCCACCAGCTGTTCCTGCAACGCCACCAGACCATCGATCCGTGCCT

*cadR*

GCACATGCTCGATATGCTCGTCGATCAGCGCATTGACGCTGCCGCACGAATCATCGGGGCTGTCGCGCAGGCGTAGCAGGCTGCGGATTTCATCCAGGGTCATGTCCAGGGTGCGGCAGTTGCGGATGAAGGTAAGCCGCTCGACGTGGGCCTGGGTGTACAGCCGGTAGTTGCCGTCGCTGCGTGCCGGCTCCGGCAGCAGCTGTTCACGCTCGTAGTAGCGGATGGTTTCCACGGCGCAGTCGGTGGCTTTGGCCAGTTCTCCGATCTTCATCACGAAATTCTCCAGCA

Divergent *cadR* promoter region

AGTGGCTTGACCCTATAGTGGCTACAGGGTGTTCACTTGGCAACAGGCTCAAATTAAGGATGACCCC**TCTAGA**AATAATTTTGTTTAACTTTAAGAAGGAGATATACATATG—*mcherry*--TAA**CTCGAG**CACCACCACCACCACCACTGAGATCCGGCTGCTAACAAAGCCCGAAAGGAAGCTGAGTTGGCTGCTGCCACCGCTGAGCAATAACTAGCATAACCCCTTGGGGCCTCTAAACGGGTCTTGAGGGGTTTTTTG

**pCadC-G-CadR-R**

**Fig. S1** The cloning/expression region of recombinant plasmids used in this study. DNA sequence and annotation data are all marked.


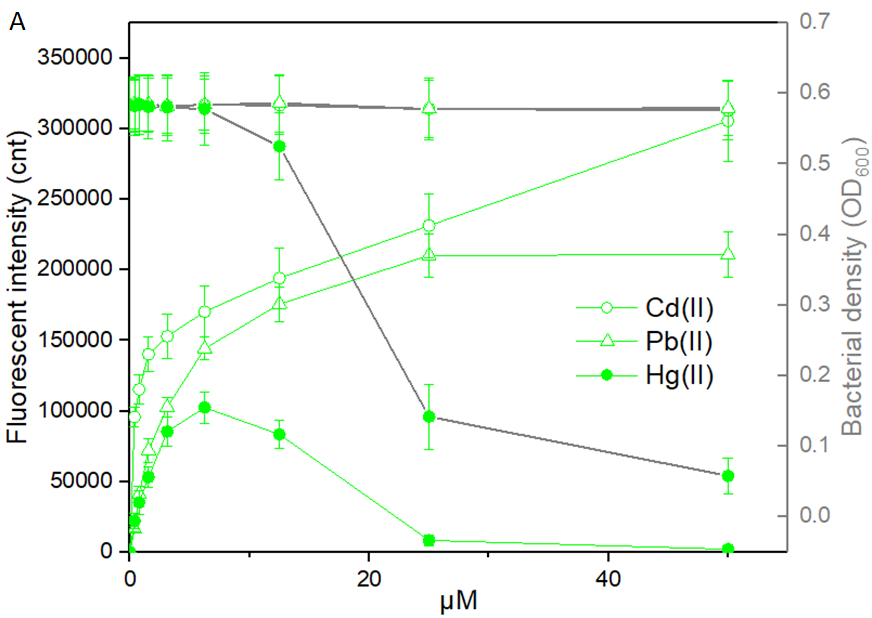


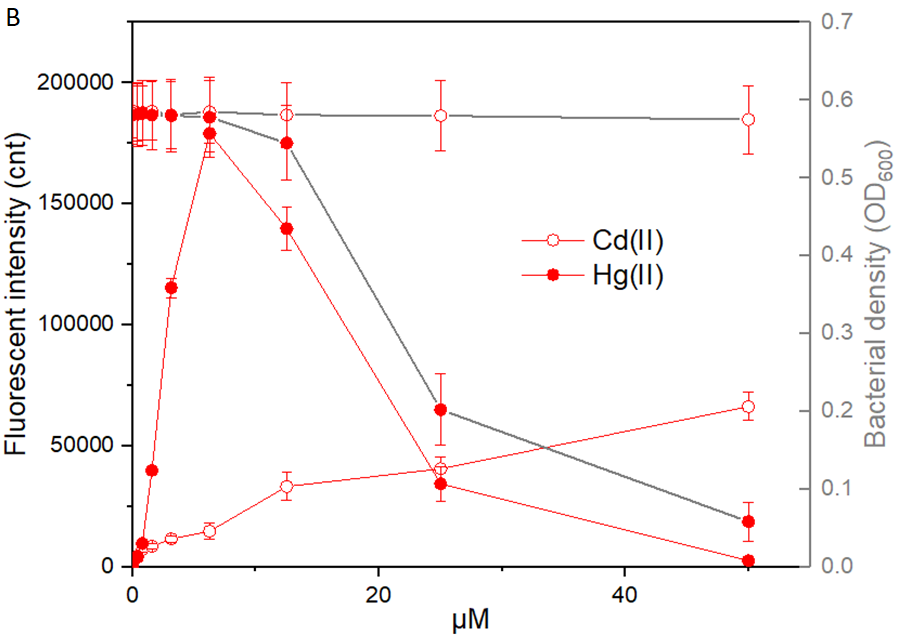


**Fig. S2** Response curves of two separate whole-cell biosensors (A) TOP10/pCadC-G and (B) TOP10/pCadR-R in response to target Cd(II) and in response to non-target metal ions.

Lag phase of TOP10/pCadC-G was exposed to gradient concentrations of Cd(II) (open circle), Pb(II) (open triangle) or Hg(II) (closed circle) generated from a double dilution method. Lag phase of TOP10/pCadR-R was exposed to gradient concentrations of Cd(II) (open circle) or Hg(II) (closed circle) generated from a double dilution method. After a 12-h incubation at 37^o^C, bacterial cell density was measured (grey line diagram, right-Y scale), and both eGFP and mCherry fluorescence were determined (red or green line diagram, left-Y scale). Results are the average of at least three independent experiments performed in triplicate. The fluorescent signal was indicated as a fluorescence count value (unit = cnt), and fluorescent values were divided by the optical density at 600 nm. The data values shown for each metal exposure group were obtained by subtracting the control values (with no metal exposure) from the experimental values.


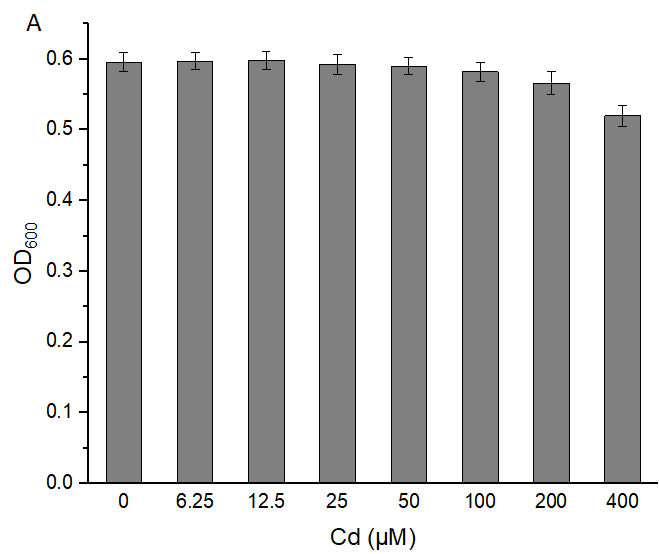


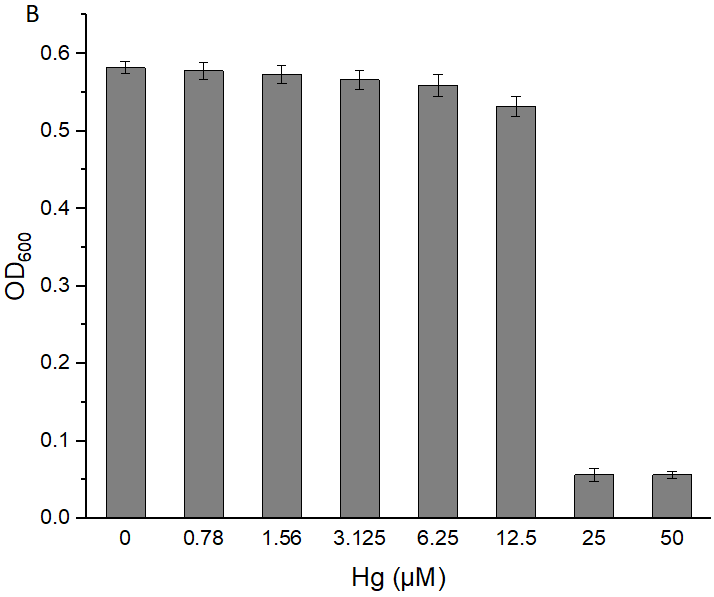


**Fig.S3** Toxic effects of Cd(II) and Hg(II) on the growth of the host TOP10.

(A) Lag phase of *E. coli* TOP10 were exposed to a gradient concentration of Cd(II). (B) Lag phase of *E. coli* TOP10 were exposed to a gradient concentration of Hg(II). After being cultured at 37^o^C for 12 h, the absorbance of each culture was determined at 600 nm.


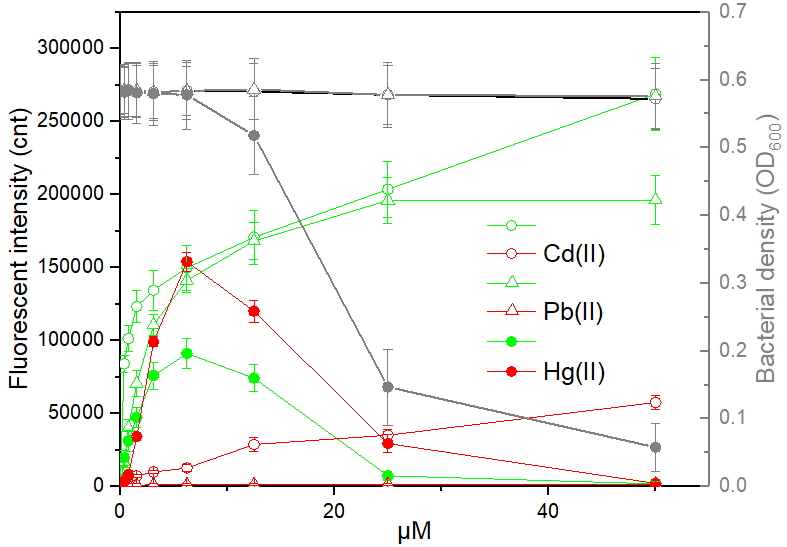


**Fig. S4** Response curve of the dual-sensing biosensor cell in response to target Cd(II) and in response to non-target metal ions.

Lag phase of TOP10/pCadC-G-CadR-R was exposed to gradient concentrations of Cd(II) (open circle), Pb(II) (open triangle) or Hg(II) (closed circle) generated from a double dilution method. After a 12-h incubation at 37^o^C, bacterial cell density was measured (grey line diagram, right-Y scale), and both eGFP and mCherry fluorescence were determined (red and green line diagram, left-Y scale). Results are the average of at least three independent experiments performed in triplicate. The fluorescent signal was indicated as a fluorescence count value (unit = cnt), and fluorescent values were divided by the optical density at 600 nm. The data values shown for each metal exposure group were obtained by subtracting the control values (with no metal exposure) from the experimental values.


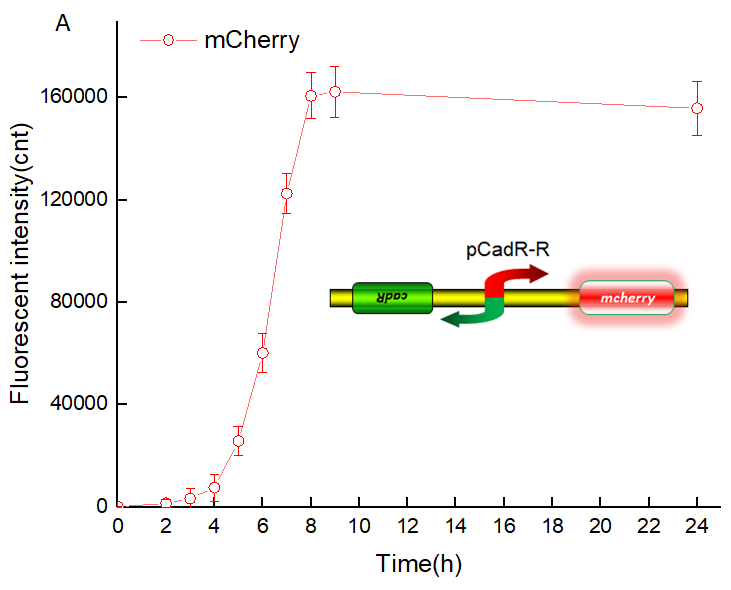


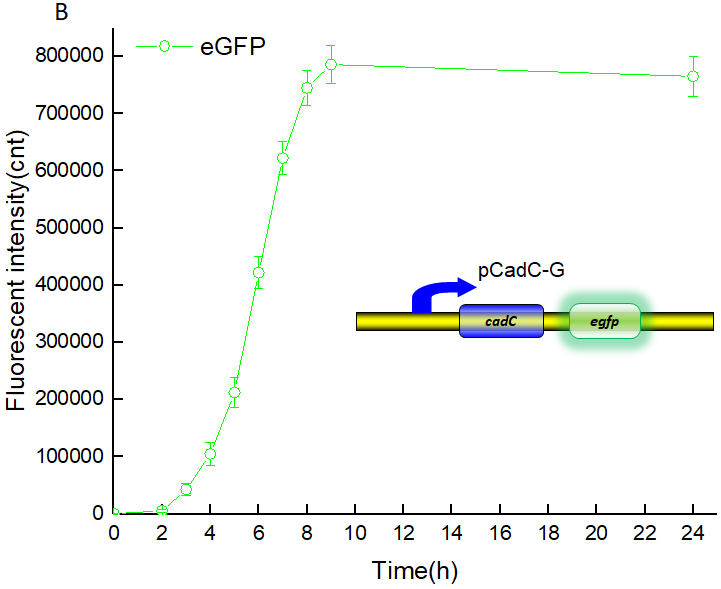


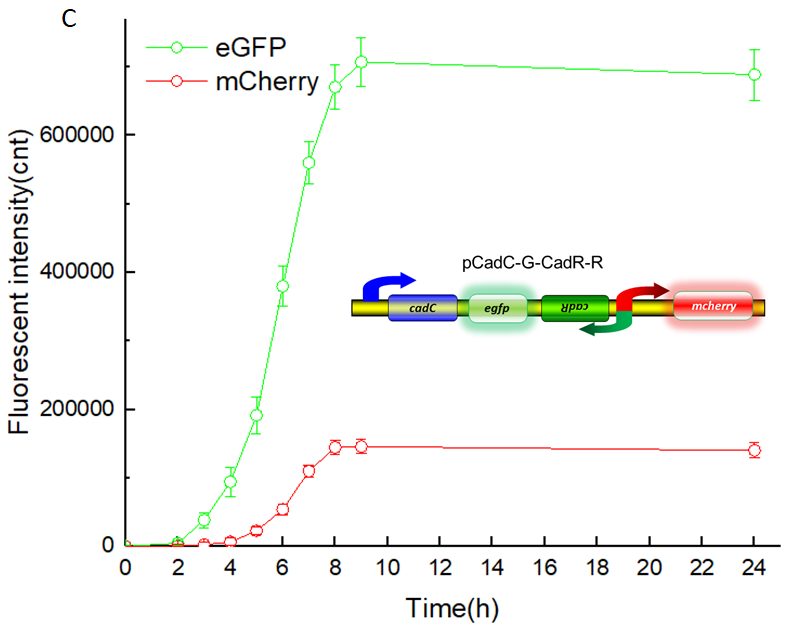


**Fig. S5** Time courses of fluorescent signals generated by three whole-cell biosensors with 100 μM Cd(II) exposure.

(A) Lag phase cultures of TOP10/pCadR-R, (B) TOP10/pCadC-G, and (C) TOP10/pCadC-G-CadR-R were exposed to 100 μM Cd(II) at 37^o^C. Two kinds of fluorescent signals were determined at regular time intervals. A 100 μL aliquot of the culture was transferred into a 96-well microplate, and the optical density of each well was measured at 600 nm. Both fluorescent signals were normalized to bacterial cell densities at 600 nm. The fluorescent signal was indicated as a fluorescence count value (unit = cnt). The results are shown as the mean of three independent assays ± the standard deviation.
